# Behavioral anatomy of a hunt: Using dynamic real-world paradigm and computer vision to compare human user-generated strategies with prey movement varying in predictability

**DOI:** 10.3758/s13414-020-02016-z

**Published:** 2020-05-13

**Authors:** Shaktee Sandhu, Tauseef Gulrez, Warren Mansell

**Affiliations:** 1 CeNTrUM (Centre for New Treatments and Understanding in Mental Health), Division of Psychology and Mental Health, School of Health Sciences, Faculty of Biology Medicine and Health, University of Manchester, Manchester Academic Health Science Centre, 2nd Floor Zochonis Building, Brunswick Street, Manchester, M13 9PL UK; 2 School of Computing, Science and Engineering, Salford Innovation Research Centre (SIRC), Autonomous Systems and Robotics, University of Salford, Salford, M5 4WT UK

**Keywords:** Prediction error, Computer vision, Ethology, Human performance, Cybernetic, Perceptual control theory

## Abstract

It is commonly thought that the mind constructs predictive models of the environment to plan an appropriate behavioral response. Therefore a more predictable environment should entail better performance, and prey should move in an unpredictable (random) manner to evade capture, known as protean motion. To test this, we created a novel experimental design and analysis in which human participants took the role of predator or prey. The predator was set the task of capturing the prey, while the prey was set the task of escaping. Participants performed this task standing on separate sides of a board and controlling a marker representing them. In three conditions, the prey followed a pattern of movement with varying predictability (predictable, semi-random, and random) and in one condition moved autonomously (user generated). The user-generated condition illustrated a naturalistic, dynamic environment involving a purposeful agent whose degree of predictability was not known in advance. The average distance between participants was measured through a video analysis custom-built in MATLAB. The user-generated condition had the largest average distance. This indicated that, rather than moving randomly (protean motion), humans may naturally employ a cybernetic escape strategy that dynamically maximizes perceived distance, regardless of the predictability of this strategy.

## Introduction

Understanding the mind has been at the center of psychology since its inception (Dewey, 1887; Edward, 1896; Wundt, 1859), yet we are still uncertain how it gathers and disseminates information (Ransom et al., 2017). One strategy is that the brain is a hypothesis prediction testing system. Suppose that you are pursuing an object and your cognitive system wants to gather information about the object from the sources you have available. Rather than being informed by the sources, the system will inform the sources of what it already thinks the object’s parameters are. The sources responds with how these predictions are false, resulting in a prediction update if this is the case. This update takes into consideration the success or failure of previous predictions in that context. This is known as the Prediction Error Minimization Theory (PMT) (Hohwy, 2013). Prediction can be seen here as the core component of this theory and the feature that unifies it with the previous literature: theory of mind (Hiatt & Trafton, 2010), oculomotor control (de Xivry et al., 2008), and visual perception learning (Stefanics et al., 2014).

Prediction is advantageous when the situation is temporally sensitive and a neurological delay in transmitting information may be costly, and more so when prior predictions are highly accurate (Ransom et al., 2017). However, prediction can be maladaptive when the environment is unpredictable or at the very least difficult to predict. This is a fundamental limitation within the PMT given the environment contains both predictable and unpredictable events (Phillips, 2001). In such an environmental context, it may be more adaptive for both predator and prey to follow a cybernetic control strategy regardless of the predictability of the movement. One possibility is that the prey would attempt to maximize distance whereas the predator would attempt to minimize distance. There is convergent evidence that, in contrast to predictive accounts, this simpler model may apply to animal behavior, such as robbing and dodging in rats and crickets (Bell et al., 2015).

We sought to develop an experimental paradigm that assessed the role of the predictability of the environment in performance. We selected the predator–prey scenario because it is a complex, naturalistic activity that is analogous to other real-world scenarios (e.g., team sports), and can be studied in the laboratory. Researchers have begun to employ laboratory experiments rather than field observations when studying predator and prey behavior (Beauchamp et al., 2007). Secondly, some researchers have begun to move away from investigating the pursuit strategies of animals and have instead investigated the behavior of humans in comparable contexts (Shaffer et al., 2013). The reason being, humans can receive instructions and manage their behavior far better than animal, allowing it to be represented within a 2D environment making the analysis more reliable and modeling more accurate (Jones et al., 2011).

Amongst the animal studies of predator–prey behavior, a frequently documented phenomenon is the erratic evasive behavior exhibited by prey during a pursuit. This behavior was termed “protean” by Chance and Russell (1959) and Humphries and Driver (1967) to classify the erratic escape trajectories exhibited by prey to evade their pursuer. Although protean behavior is highly variable in nature it is not entirely random; it sometimes even benefits the prey to move towards the predator and close the distance, making it easier for the prey to monitor the predator and/or make it more difficult for it to launch an attack (Eilam, 2005; Godin & Davis, 1995). Many animals including ducks, rats and crickets have been shown to display protean behavior (Humphries & Driver, 1967). It is commonly thought that the unpredictability of protean behavior makes it adaptive. However, the relationship between the predictability of prey movement and capture is complex. For example, a study of jumping spiders found that the predictability of prey movement only benefited predators with a ‘docile personality’, whereas prey with unpredictable movements were better captured by predators with an ‘aggressive personality’ (Chang et al., 2017).

It is also the case that prediction only benefits the prey within a certain range of relative speeds of the predator and prey. Specifically, if the prey is significantly faster than the predator, then it can easily evade capture. “In the absence of the prey turning or exploiting features of the landscape inaccessible to predators, the predator must simply maintain a speed greater than the prey for a sufficient time to successfully catch the prey” (Moore & Biewener, 2015). For example, naturalistic studies of animals show that some prey can often simply outrun predators to evade capture (Combes et al., 2013; Moore & Biewener, 2015).

We have identified three studies that have explored the strategy that human participants take in predator–prey scenarios. Jones et al., (2011) investigated the effect a predictable pattern of movement from prey has upon a predator. They investigated whether grouped prey were less easily captured with protean behavior compared to predictable behavior. This was investigated by human participants controlling a digital predator and attempting to capture computerized targets with protean or predictable patterns of movement. Targets with predictable behavior were more likely to be captured than those with protean behavior. However, there is evidence that the relationship is more complex in situations in which the prey move in groups, as is commonly the case in nature. In a study of human participants pursuing prey on a computer, prey movement unpredictability interacted synergistically with the density of the group to reduce capture performance (Chang et al., 2017). The advantages of protean behavior also appear to be related to complexity in recent study that instructed human predators to track prey in three-dimensions using their head movements within virtual reality (Richardson et al., 2018). This study revealed that protean movement reduced predator accuracy only when combined with predictable movements to generate more mathematically complex trajectories. Furthermore, even complex movement trajectories (e.g., spirals) that were nonetheless mathematically predictable, reduced predator accuracy.

Most importantly for our aims, none of the above studies included a benchmark of human prey to establish whether the degree of unpredictability is the feature that distinguishes the evasion of capture in real agents. Our study included a number of novel methodological components to advance on the earlier literature: human participants in both the predator and prey roles; a naturalistic design using a hexagonal grid for the pursuit; computer vision (CV) analysis to establish movement trajectories.

In CV, dynamic object detection in real-world dynamic environments is a very difficult problem to solve, as many different parameters are required to be reconciled in one algorithm (Moeslund and Granum, 2001). For example, a detector capable of handling continuously varying illumination may find it difficult to cope with target’s continuously changing appearance due to variations in viewpoints (Cucchiara et al., 2003). In order to detect and track both prey and predator’s movements in a fast-changing dynamic environment, we developed a detector which considers the distinction of a foreground against the background. The target can be modeled as a stand-alone entity (while not as its distinction from the background), changing effect of light or change of viewpoint can invalidate the target model (Smeulders et al., 2013). Hence, in this study a tracking methodology is developed for the detection of the target using foreground/background color and intensity discrimination. A discrimination function is trained for quantification of foreground/background discrimination.

The aim of the current study was to investigate whether the predictability of a target’s movement has an effect upon a pursuer’s pursuit strategy. Furthermore, it aimed to investigate how this compares to the pursuit strategy used naturally by humans to pursue another human (user generated). This was investigated by comparing the average distance between individuals in a natural pursuit to benchmarks of distance when the target’s (prey) movements vary in predictability: from predictable, to semi-random, to random. We hypothesized a negative linear relationship between distance and prey movement predictability. We planned to compare the user-generated condition to these to judge whether naturalistic prey movement is likely to adopt the strategy of unpredictable movement and achieve predator–prey distances approaching the random condition. Alternatively, if the human prey is superior to all the pre-programmed conditions varying in predictability, then a dynamic control strategy may be used that maximizes distance from the predator, over and above any advantages of making unpredictable movements.

## Method

### Design

A pair of participants was randomly allocated to their role of predator or prey and completed a series of pursuit and evasion tasks. The interaction between participants was video recorded for later analysis. The design was a repeated-measure one-way ANOVA with the independent variable being the prey participant’s pattern of movement. This consisted of four levels: predictable (P), semi-predictable (S), random (R) and user generated (U). The conditions were counterbalanced as such: PSRU, UPSR, RUPS, SRUP. The dependent variable was the mean distance between the participant’s markers (laser and magnet) in each condition.

### Participants

Forty participants (25 males, 15 females) were recruited through opportunity and volunteer sampling at the University of Manchester.

## Materials

### Piloting - board

Piloting the experiment resulted in several changes to the design of the apparatus and procedure. Firstly, the landscape/map in which participants would have interacted upon was initially a large circle with a radius of 20 cm made up of hexagons with a diameter of 1.5 cm. The prey and predator’s starting position was at the center of circle at 2 hexagons apart. The prey were instructed to reach the edge of the circle to escape successfully, while the predator was instructed to pursuit and catch the prey by keeping as close as possible. This test exposed two limitations of this design: one, the escape space available to the predator was limited and, two, the magnets representing each participant interfered with each other. As a result of the circular map, the prey’s escape trajectories were limited to a 180° area away from the predator’s starting position. Furthermore, the prey trajectories were increasingly limited given that the risk of being caught increased the closer the escape trajectories approached 90° to the predator’s position. Consequently, a circular map was abandoned in favor of a rectangular map whereby the predator and prey started on the left side of the map and traversed across. The prey reaching the right side of the map was considered a successful escape and end of the trial.

### Board

A wooden frame (94 × 61 cm) was built with a slot for two transparent Perspex sheets in order to enclose the double-sided hexagonal graph paper placed within the frame (see Fig. 2). This will be referred to as the board from here onwards. The board was placed on top of a table to allow the participants to interact while standing.

### Piloting – trajectory patterns

One of the pre-determined patterns of movement was a sinusoidal wave form (predictable), however, piloting revealed that varying the frequency and amplitude significantly affected the predator’s ability to extract the predictable pattern wave form. Given the landscape participants interacted upon was made up of hexagons the sinusoidal pattern conformed to this hexagonal configuration, in other words, the wave form was not mathematically sinusoidal but an imitation with straight lines. Nonetheless, piloting found that a sinusoidal wave with a frequency that was too small or too large impeded upon the prominence of the sinusoidal wave form, putting the validity of the predictive condition at risk. Piloting revealed an optimal sinusoidal wave frequency of 0.19 Hz with an amplitude of 7.5 cm (2.5 hexagons).

### Trajectory patterns

**Fig. 1:**
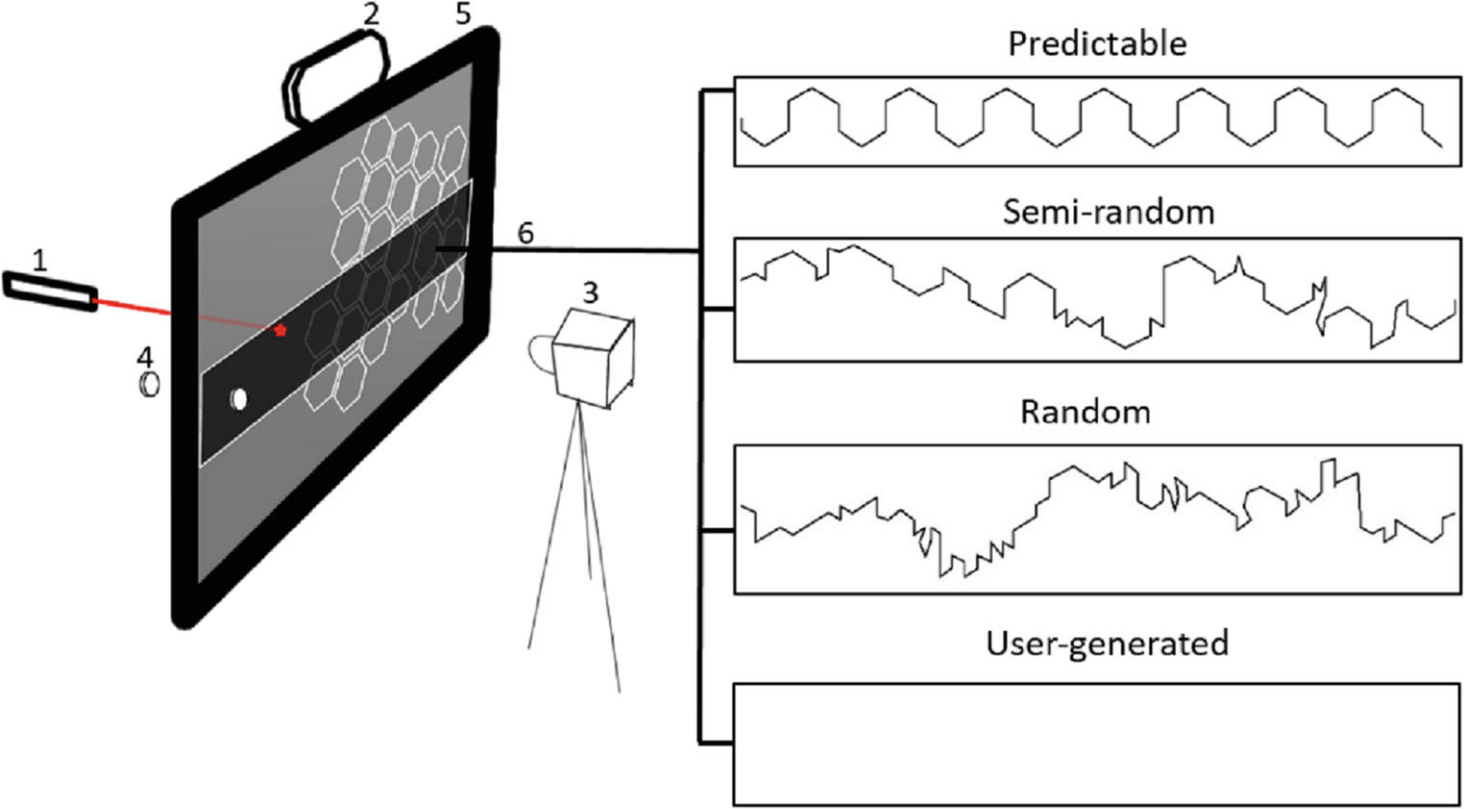
Experimental setup and four trajectory patterns. 1 = Laser pen, 2 = Metronome, 3 = Video camera, 4 = Magnets and 5 = Board

Three strips of clear acetate were constructed with pre-determined patterns of movement printed onto them with varying degrees of predictability: predictable, semi-random, and random. Each strip was placed individually, according to their respective condition, upon the hexagonal graph paper at a standardized location, resting between the hexagonal graph paper and Perspex sheet (see Fig. 1).

### Piloting – participant representation

Initially, each participant had a magnetic stylus to control their magnet on the opposing side of the board. However, at a minimal distance the magnet of the opposing participant would become attracted to the incorrect magnetic stylus. In other words, the predator participant’s magnetic stylus would become attracted to the prey participant’s magnet which lies on the predator’s side of the board and vice versa. Therefore, the prey’s representation on the board became a laser point emitted from a laser pen. Only the prey’s representation was changed to allow for a clear differentiation between the predator and prey.

Secondly, using a laser point to represent the prey although overcome one limitation introduced another; an unfair advantage of speed. The prey participant’s laser point was capable of traveling a greater speed than the predator’s counterpart. As a result of the laser pen’s light weight and small size, it had considerable maneuverability, whereas the friction from the magnet and magnetic stylus against the board limited the predator’s speed. In order to overcome this limitation the speed of both the prey and predator were standardized with a metronome, allowing both participants to travel at an equal speed. The optimal metronome setting was 70 bpm (beat per minutes). A lower bpm allowed participants an excessive amount of time to consider their next position in relation to the other participant; lacking ecological validity of natural pursuits. A higher bpm inhibited participants traveling at one hexagon per beat, resulting in unstandardized speeds and deviations from the pre-determined patterns of movement.

### Participant representation

**Fig. 2:**
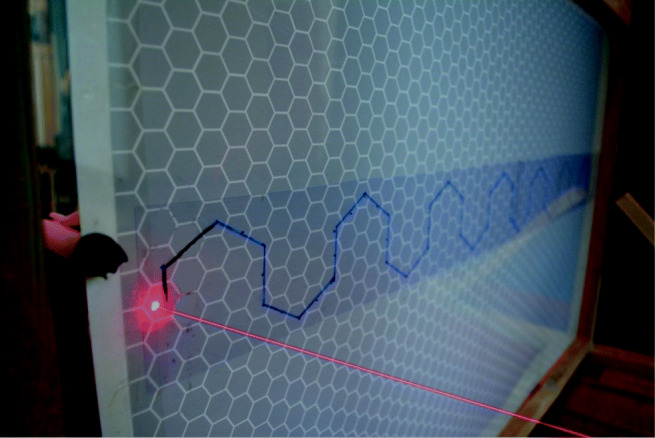
The prey’s side of the board displaying the prey’s laser point, predictable pattern of movement and the predator’s corresponding black magnet

A magnetic stylus and magnet were provided for the predator participant and a laser pen was provided for the prey participant (see Figure 2 and Appendix). A digital metronome set at 70 beats per minute was used in order to standardize the speed of each participant’s movement (see Fig. 2).

### Videoing equipment

A Nikon D5200 DSLR (digital single-lens reflex) camera was placed at a distance of 110 cm perpendicular to the center of the board and recorded at 50 frames per second (image resolution 1280 × 720) the participants’ interaction on the board (Fig. 1).

## Tracking software development

Before we explain the development of tracking software, we want to mention that a MATLAB function ‘hex-grid-generator’ was developed and made publicly available on https://www.mathworks.com/matlabcentral/fileexchange/67680-hex_grid_generator. The version of this function fall under an open-source GNU general public license (GPL). Scientists can contact author TG or WM to obtain the executive file of the full-software to test on videos recorded under variable conditions. This software function and executive file of full software remains intellectual property of TG and WM and it will not be published on any other websites without permission.

The extraction of the prey/predator movements from trial videos requires a computer vision (CV) software. The CV software development relies on a series of image processing and CV based steps to extract information from the recorded videos in order to determine prey/predator movements. These steps consisted of (i) spatiotemporal cropping of videos, (ii) unsupervised detection of the centers of hexagonal grid points on the board, (iii) motion tracking of prey/predator movements, (iv) trajectory formation of movement points for both the prey and the predator. Here we provide detailed information per individual processing step.

### Spatiotemporal cropping

A spatiotemporal cropping of video frames was required in order to remove clutter and color artifacts, e.g., brown color of wooden border, light reflections, etc. Therefore each video was cropped to a fixed region of interest (in our case the frontal looking hexagonal grid board) and removing irrelevant background objects. Videos were also cropped in time by removing the stationary paradigms (start and end of the trial). The start of the videos often contained an increase in the prey’s movements (flickering laser pointer’s light intensity or sometimes reflections from the board’s corner) and the end tended to contain prey’s (laser pointer’s) unnecessary movements, as participants anticipated the end of recording.

### Hexagonal grid detection

**Fig. 3:**
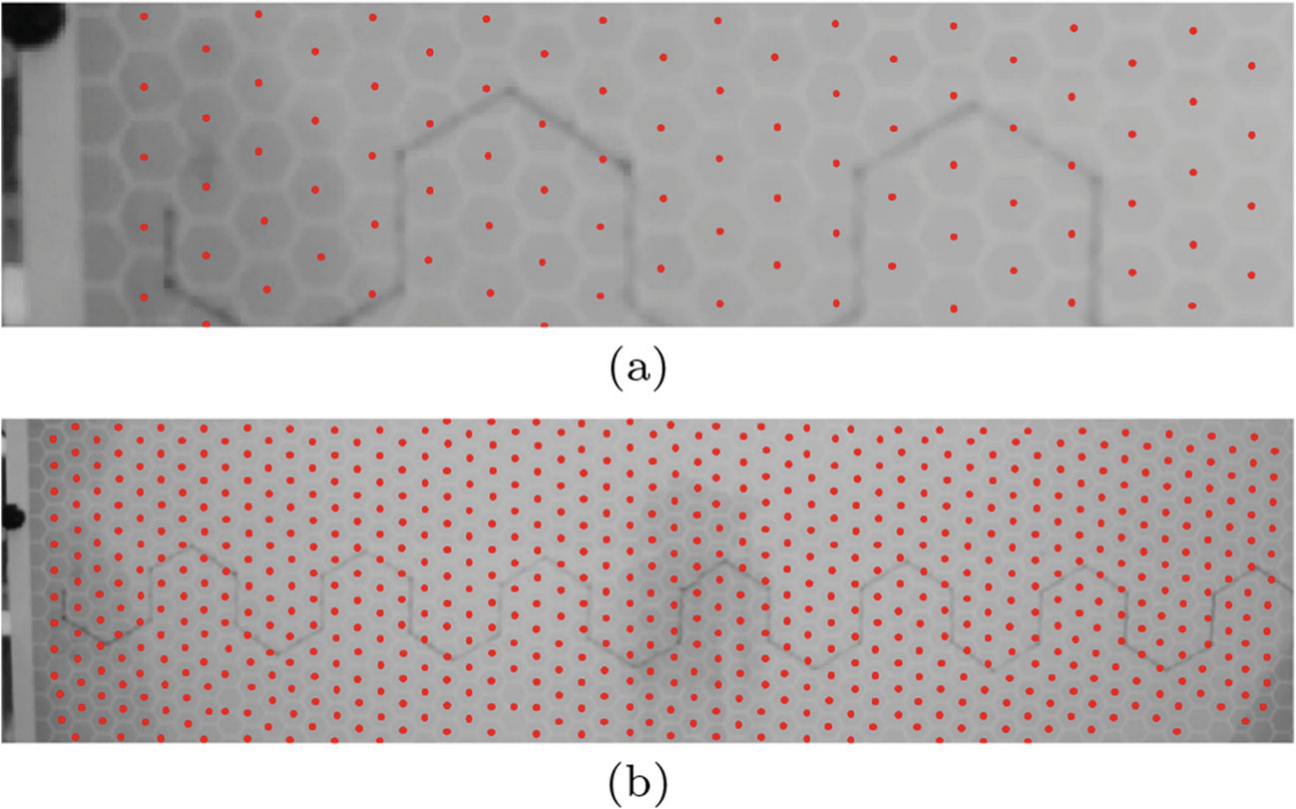
Center point of each hexagon on the hexagonal grid are detected, shown in *red*

A module was developed in our software to detect the centers of the hexagonal grid cells on the board. The hexagonal grid has identical and equidistant flat topped hexagons (cells) as shown in the Fig. 1 whose width *w* = 2 × size and height $h = \sqrt {3} \times \text {size}$, where size in our case is 1.5 cm. The edges or the line segments of the hexagonal grid were obtained using Canny’s edge detection method (Canny, 1987). The edges were traced and exterior boundaries of all the hexagons were obtained using MATLAB’s ‘bwboundaries’ function. The intersection point of all individual boundary points was taken as the center point of a hexagonal cell (as shown in Fig. 3a, b). Further, a customized function was developed to check the accuracy and consistency of the obtained center points of the hexagons. The distance of the center points of the cells to their corners at 45°, 90°, 135°, 225°, 270°, 315° was calculated and if the distance fell under unacceptable range, the points were considered as noisy and were replaced by the points consistent with the corner distance and nearest-neighbor distance to the adjacent and accurately estimated center points. This detection method worked well for all the frames of the trials and the cell points were saved as individual trial files.

### Object detection (prey/predator)

**Fig. 4:**
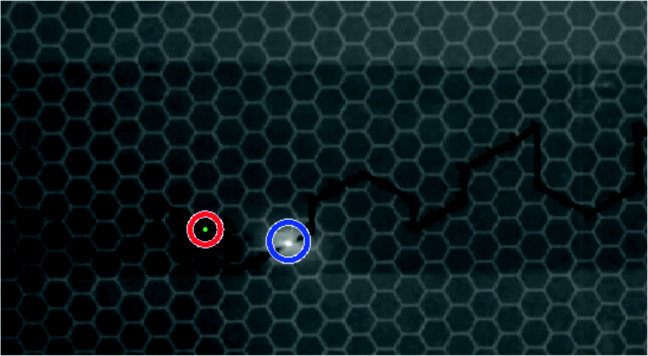
Prey and predator were detected and tracked in motion through out the trial’s videos, represented by *blue* and *red circles*, respectively

The predator (magnet) was wrapped with thick black tape making it visible as a black dot, where as prey produced a bright red light feature on the board (as shown in Fig. 2). An object detection algorithm was developed in MATLAB using image processing and computer vision system toolboxes, to detect and track prey and predator’s movements on the board during the trial. The object detection algorithm performed two processes in parallel. The first process was aimed to detect prey as a bright red object. The ‘Red’ color channel was separated from the RGB frame and a median filter (3 × 3) was applied to the resultant ‘Red’ object to remove the noise. A ‘circular Hough transform’ (Pei & Horng, 1995) (‘imfindcircles’ MATLAB function) was applied to the red object pixels and a circle was drawn (Barnstedt et al., 2015), the center point of the circle was obtained (in 2D *xy* coordinates) and were considered as prey’s movement points. The second process was designed to detect a moving black dot (as predator) on the white board. This was achieved by conversion of the frame into binary format, removing all connected components fewer than ten pixels, and later morphological close operation, i.e., dilation followed by an erosion was performed to make black dot prominent. Similar to the first process, a circle was fitted to the resultant points and the center point was obtained and tracked as predator moved on the board as shown in Fig. 4.

### Trajectory formation of prey and predator

**Fig. 5:**
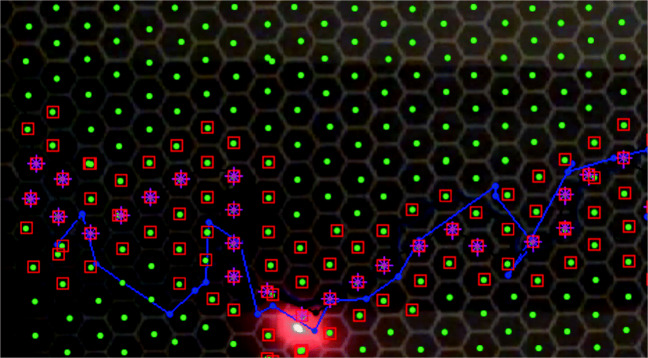
Prey and predator pursuit analysis snapshot. The *solid black line* is the prescribed trajectory. The *center points* of hexagonal grid cells are *green dots*, the *blue* and *red* trajectories are of prey and predator, respectively. The *red squares* are the possible predator movements. The *pink asterisks* are the actual predator movements

The 2D (*x*,*y*) positions of the prey and the predator for each trial were recorded. In order to map the 2D positions of the prey and predator on the grid, the movements of prey and predator in time were associated to the each hexagonal grid’s center position. For every trial, two trajectories were obtained, manifesting prey and predator movements over the course of trial (as shown in Fig. 5). The start and end frame of the trial video was selected manually, to make sure that the predator and prey were always present in the video frames (to obtain a continuity of the natural trajectories).

### Comparison of manual vs. computer vision-based trajectory extraction

**Fig. 6:**
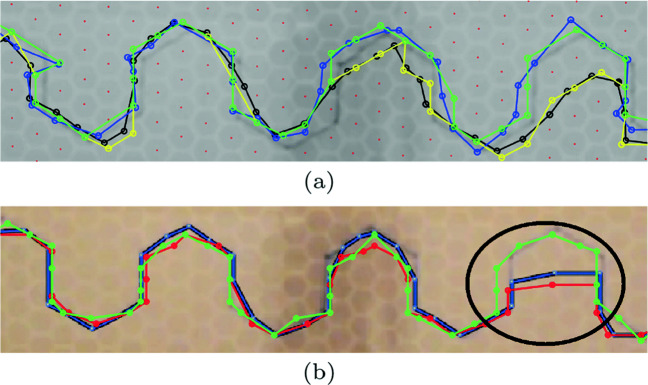
Pursuit trajectories analysis. **a** The *black* and *blue* trajectories are of prey and predator, respectively. *Green* and *yellow* trajectories are the associated trajectories with the hexagonal grid center points. The *center points* of hexagonal grid cells are *red dots*. **b** The *red* and *green* trajectories are of prey and predator, respectively. The visible trajectory erroneous area is encircled *black*. The associated true trajectory can be seen in (a)

To verify the performance of the proposed computer vision-based detector, we compared it with the results obtained by two independent coders (one author and one volunteer). The proposed computer vision method was programmed with MATLAB. All experiments were implemented on Intel Core i7-6500 CPU at 3.19 GHz and 16-GB RAM. The two coders then assessed and coded the trajectories in the video (manually, frame by frame) by applying a template and recorded *x* and *y* locations of predator and prey at each beat. Although the trajectories are subjected to error and are bias intrinsic to subjective estimation, the human coders manual results are a particularly valid comparison being a direct estimation of the target phenomena of both prey and predator detection in images. To visually compare the accuracy of our detection method and manual detections, we have shown the both trajectories in Fig. 6a, b. We observed that human coders made errors on several occasions one of which is shown in Fig. 6b. We also verified the error by visually observing the video and comparing both trajectories and came to the conclusion that computer vision-based results were more accurate. A total of 68 (*x*,*y*) points were detected for each prey and predator movements using computer vision, whereas human coder detected 59 points. It took a human coder 2 h (7200 s) to complete the task, whereas computer vision took 180 s or 3 min to complete the same task.

## Results

**Fig. 7:**
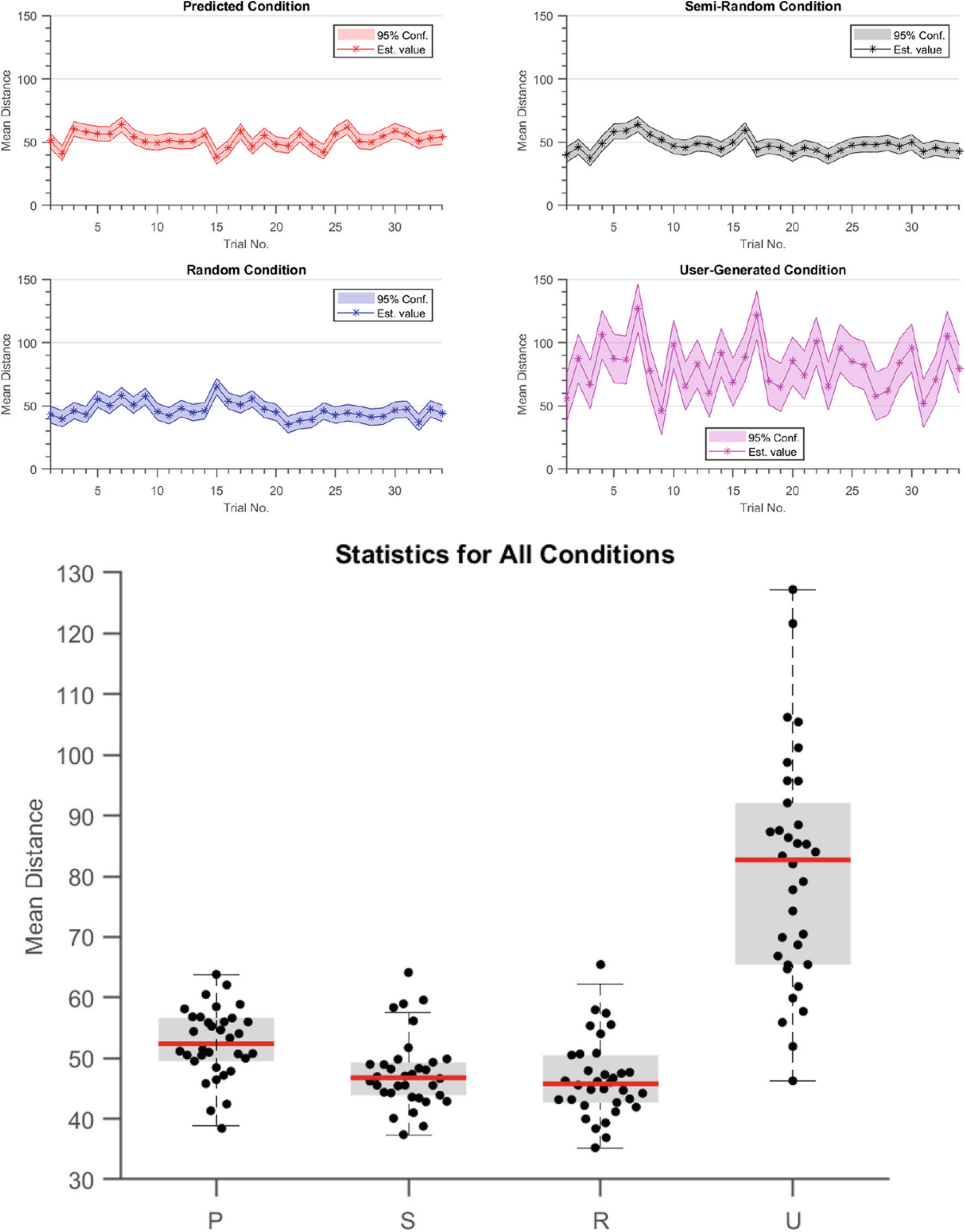
Results of the experiments. **a** The average distance between the predator and prey across the four different conditions of movement with 95% confidence intervals. **b** Boxplot for all conditions; the *red line* is mean data

In Fig. 7 descriptive statistics of the average distance between the predator and prey within each condition has been shown. A repeated one-way ANOVA (Huck & McLean, 1975) revealed a significant difference between the conditions means, *F*(1.19,34.53) = 75.2,*p* < .001 (Geisser et al., 1959; Abdi, 2010). This represented a large effect size; *η*^2^ = 0.669, revealing that 67% of the average distance between the predator and prey was accounted by the user-generated movement and varying predictability of the prey’s movement.

**Fig. 8:**
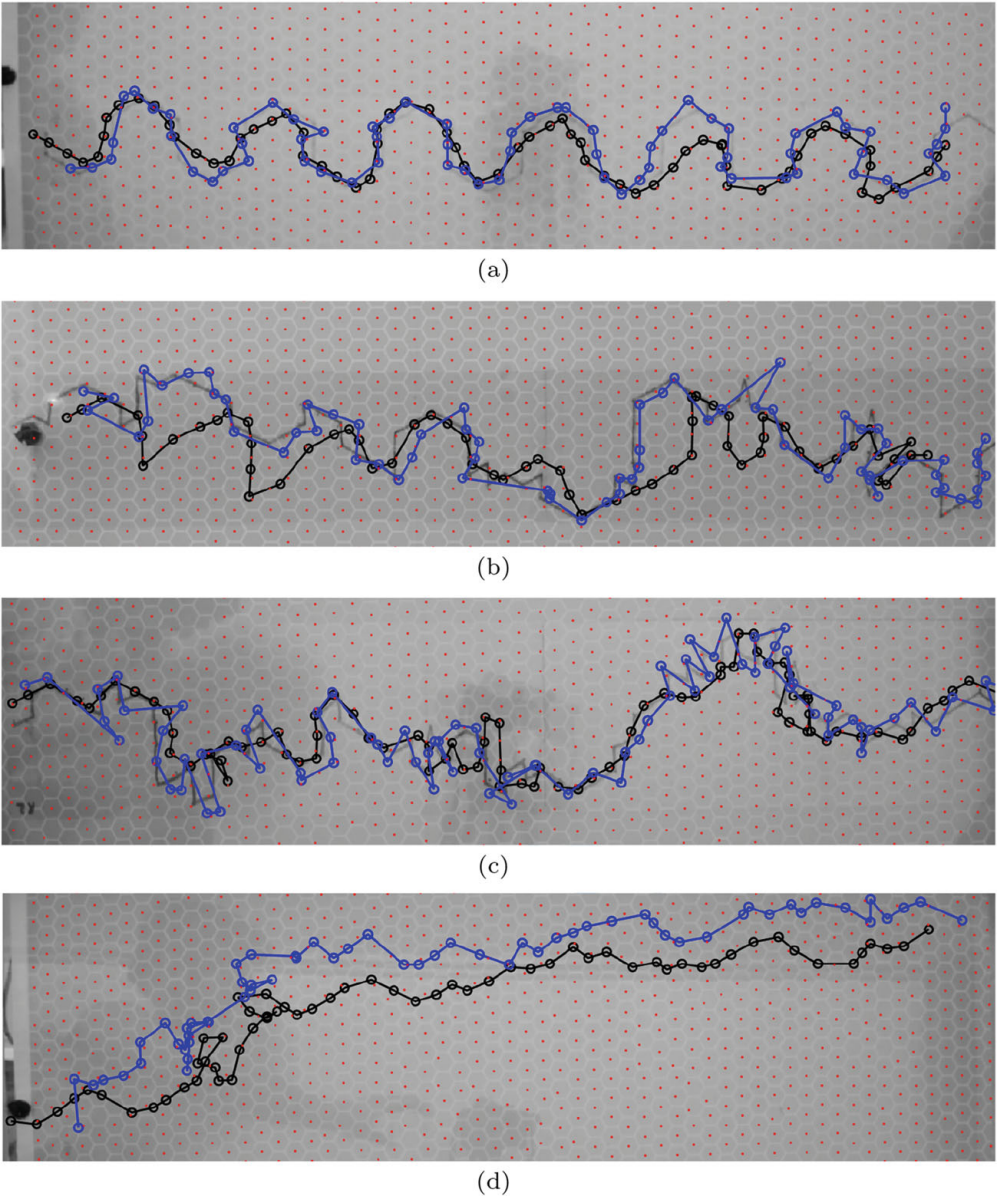
Examples of the trajectories taken by the predator (*black trajectory*) and prey (*blue trajectory*) within each condition, as well as, their relative location to each other throughout the trial. **a** P - Predictable. **b** S - Semi-random. **c** R - Random. **d** U - User generated

Planned comparisons employing a repeated measure t-test (Ruxton, 2006) with a Bonferroni correction (Sedgwick, 2012) revealed a statistical difference between the user-defined condition distances and the predictable (*p* < .001), semi-random (*p* < .001) and random condition distances (*p* < .001). These differences had large effects sizes, *d* = 2.65,2.48 and 2.25. In addition, the random and semi-random condition distances were found to be statistically different from the predictable condition distances. Small *p* values were found between random and predictable (*p* = .002) and semi-random and predictable (*p* = .001) conditions, (See Fig. 7) revealing medium effect sizes, *d* = 0.67 and 0.64. However, no statistical differences were found between the predictable and semi-random (*p* = .339) condition distances (See Fig. 7); unsurprisingly resulting in a small effect size, *d* = 0.23. The predictive condition presented the predator participant with a predictable pattern of movement, allowing the predator to intercept the prey as they traveled towards the predators headed direction (see Fig. 8a, b, c, d). We can observe within the predictable condition the predator’s pursuit trajectory interception segments are divided by tracking segments, whereby the predator simply follows the prey at a distance, but this pursuit strategy incurs a cost of having a delayed response to the prey’s change in direction (see Fig. 8a, b, c, d). This was not the case in the random and semi-random conditions, where the predictability was harder to realise and so the predator strictly chased the prey on the prescribed erratic trajectory, which resulted in a mean average distance slightly less than the predictable condition.

## Discussion

In this study, we presented the development and basic results obtained from computer vision (CV)-based software aimed to demonstrate its functionality with experimental evidence: (i) to detect accurately the prey–predator movements on the hexagonal grid cells, and (ii) to examine whether the trajectories obtained are representative under natural ambient light conditions. The adoption of CV and machine learning in behavioral experiments saves manual labor-intensive analysis time and costs associated with decode video frames. As far as we know, cost–benefit analysis and accuracy had not yet been reported by previous studies with a similar experimental setup, a simple color camera and relatively straight-forward CV algorithms. It was also found that CV can be more effective and accurate under similar lighting conditions, no reflections and similar camera positions across all the trials. Future software and experimental developments could try to cover such variabilities.

Moreover, after data analysis, this study had two key findings in relation to our hypotheses. Firstly, the results from the predictable (P), semi-random (S), and random conditions (R) showed that predictable prey were tracked at a further average distance by the human predator compared to prey with random, or semi-random movements, contrary to the idea that the human mind uses prediction to aid performance (Hohwy, 2013). Two earlier studies did not show a direct relationship between unpredictable prey movement and reduced predator accuracy (Chang et al., 2017; Richardson et al., 2018). Observations of the individual predator-prey pursuits suggest that our findings may have resulted from a ‘stalk and intercept’ strategy that may entail longer average distances as the predator follows at a distance, and yet may result in successful ‘capture’ of the prey.

In contrast, the second, and most pronounced finding was that the user-generated condition produced significantly larger distances between the predator and prey than all other conditions. Human prey appeared to operate in a far more efficient manner than pre-programmed, or randomly generated, patterns of movement. Human prey may therefore have utilized an awareness of their position, relative to the predator’s position, which allowed them to make trajectory choices impromptu, thereby significantly increasing the distance between themselves and the predator. This is a cybernetic strategy (Bell & Pellis, 2011). This finding appears to undermine the importance of whether the prey movement is predictable, semi-random or random and it adds an additional factor to those of group size, complexity and speed that have been studied to date (Chang et al., 2017; Richardson et al., 2018). The predator’s ability to use prediction to catch the prey is undermined by a cybernetic prey, and yet the predator itself can use a similar approach – to minimize its distance dynamically, when the predator has control of a variable that he or she perceives i.e. predator–prey distance. In other words, it is the perceived aspect of the individual and its environment that is controlled (Powers, 1973) in both cases.

Ultimately, a model is needed that accounts for both the stalking of more predictable prey by the predator, and the advantage of a human prey’s control strategy. It is clearly feasible that the predators in the study could have attempted to predict the prey’s next move by inferring which way it would go to maximize its distance. Yet, the finding that human prey kept further away than any of the pre-set movements, indicates that if the human predators could do this prediction, they did not do it to their advantage in this study.

Cybernetic control as described by perceptual control theory can involve tracking not only the perceived position of an object, but also its current perceived velocity and direction (Marken & et al. 2001). To the extent that the current velocity of a prey in a certain direction stays the same over time, then due to its momentum, it will permit the predator to be in the correct position to get closer to the prey in the near future. This does not mean that from an internal perspective, the animal is predicting the position of the prey in the near future. It is merely tracking the prey’s current perceived relative velocity and direction. However, to the observer, this will look like prediction. Furthermore, agents can also attempt to control for the current pattern of movement that they perceive, such as a sine wave or a circle, which can also appear to be making a prediction because the current perceived pattern unfolds over time through the movement of the tracked object (Powers et al., 1960). This would be more evident for our human participants because they had (unlike real animals in current pursuit) a ‘helicopter perspective’ of themselves (the predator) and the prey in motion.

The above account leads on to the range of factors that would make the experimental testing more naturalistic. First, would be to model the perceptual field of the animals ‘on the ground’ rather than from above. When doing so, it appears that certain predators may move to keep highly specific perceptual variables constant; for example, bats keep their perception of a mantis at a fixed angle as they shorten their distance from it (Ghose et al., 2009). The second advance would be to model the physics of real predators and prey, which in turn helps to incorporate information about their relative speeds in different environments, and their abilities to change direction. Third, future studies may need to account for multiple strategies for both predator and prey, and how they are applied to the context, and learned. For example, the sudden evasive darting of certain prey may be an attempt to escape capture by eliminating the ‘looming’ perception of the predator as it attacks (Kane et al., 2015). The predator, on the other hand, as discussed above, may also sometimes control for perceptual variables that unfold over time, such as the pattern of movement of the prey, resulting in an apparent prediction of its future location. Thus, any overarching model of predator–prey pursuit is likely to be multi-layered, embodied, and have dynamically adjustable, learnable strategies and parameters.

The next stage in this research plan is to construct computational models of individual predators and prey by training them on the human performance, and testing them against new pursuits. This methodology would allow a robust test of the model fit between an agent utilizing a predictive strategy with one using a cybernetic, or perceptual control, model (Mansell & Huddy, 2018).

## Data availability

The datasets generated during experiments and analyzed for the present study are available from the corresponding author. The videos generated during the present study are not publicly available due to privacy and ethical issues.
